# Demonstrating trends for early colorectal cancer incidence: a retrospective analysis of patients in Northwest Ohio

**DOI:** 10.3389/fonc.2026.1712650

**Published:** 2026-04-20

**Authors:** Dean E. Watkins, Sonali Doshi, Luke Roberts, Claire Barker, Mohammad H. Rashid

**Affiliations:** 1 Department of Medicine, Indiana University School of Medicine, Indianapolis, IN, United States; 2 College of Medicine and Life Sciences, University of Toledo, Toledo, OH, United States; 3 Department of Hematology/Oncology, Toledo Clinic, Toledo, OH, United States

**Keywords:** colorectal cancer, early-onset cancer, hypothesis forming, midwest and great lakes, retrospective chart review

## Abstract

Colorectal cancer (CRC) is one of the most prevalent malignancies in the world and is the second leading cause of cancer mortality. Concerningly, CRC affecting patients aged 30–49 has been skyrocketing in recent decades, which has prompted adjustments to screening guidelines in the United States in 2023. With this single-center retrospective chart review, we evaluated 230 patients with CRC from the Northwest Ohio region with the goal of potentially identifying trends in demographic, lifestyle, and molecular risk factors in these patients. With this study, we believe that we have produced a data set that, while limited by a small early-onset cohort and unadjusted analyses, may be appropriate for use as a hypothesis-generating tool for future studies within the region in order to help direct further discussion regarding CRC in patients aged 30–49.

## Introduction

Colorectal cancer (CRC) refers to malignancy affecting the colon, rectum, and excludes the anus, small intestine, stomach, and esophagus (1). CRC develops from neoplastic growth of the colorectal mucosa, which may originate from various sources, including hyperplasia, atypical hyperplasia, adenomas, and other neoplastic proliferations (1). Globally, CRC is the third most commonly diagnosed cancer and the second leading cause of cancer-related death (1). In the U.S. alone, 2024 saw an estimated 152,810 new cases and approximately 53,010 deaths from CRC, accounting for 8.7% of all cancer deaths (2). The lifetime risk of developing CRC is about 1 in 24 for men and 1 in 26 for women (3). The highest burden in the U.S. is concentrated in the Southern, Midwestern, and Appalachian regions, with Mississippi showing the highest incidence at 46.5 per 100,000 people (4).

The number of colorectal carcinogenesis pathways is vast. The most common being the adenoma-carcinoma sequence (5). This sequence begins with genetic mutations that drive the formation of an adenoma. Over time, further mutations result in increasing dysplasia, ultimately leading to carcinoma (5). It is important to note that not every adenoma will progress to carcinoma. Approximately 5% of all adenomas progress to cancer (6). This is partly due to the fact that CRC development typically does not follow a single genetic mutation. Instead, multiple genetic pathways are often implicated. One particularly interesting method of CRC development is the interplay between microsatellite instability and mismatch repair (MMR). Microsatellites refer to naturally occurring repeated DNA bases within specific genomic regions (7). These sequences are unique to an individual and act as a genetic thumbprint. Despite their importance, these repetitive regions are surprisingly prone to error. In genetics, this paradoxical process is referred to as microsatellite instability (7). Microsatellite instability can have a dramatic effect on the function and wellbeing of a cell. Normally, MMR enzymes correct these errors, but when MMR fails—due to either inherited or sporadic mutations—MSI can occur, leading to unchecked cellular proliferation and adenoma formation (5, 7). Errors in the genetic coding for mismatch repair proteins can lead to an ineffective MMR process. These errors may be traced back to an inherited genetic source. For example, familial CRC has been linked mismatch repair genes such as MLH1, PMS1, and PMS2 (7). Additionally, MSH2, MLH1, MSH6, and PMS2 are supported variances found in individuals affected by Lynch syndrome (7). Sporadic errors may also result in disruption of the MMR process. Research supports a wide variety of sporadic errors including but not limited to; methylation of CpG islands on genetic sequences coding for MMR proteins, absence of promoters, and RAS/MAPK signaling abnormalities such as BRAF V600E (7). The product of the oncogene, *BRAF*, is a serine/threonine protein kinase that plays a critical role in directing cell proliferation, differentiation, migration, and death, in addition to angiogenesis via the mitogen-activated protein kinase (MAPK) signaling cascade (8). Other key genes whose mutations are involved in CRC tumorigenesis include: *KRAS* and *NRAS*, oncogenes whose products ultimately regulate cell growth and differentiation via their GTP-dependent activity; *TP53*, tumor-suppressor gene whose product is famously responsible for regulating DNA repair, cell cycle arrest, and cell death in response to upregulation and phosphorylation following DNA damage; and most importantly *APC*, a tumor-suppressor gene whose product interacts with several other regulatory proteins to moderate chromosome segregation, cell migration and adhesion, as well as cell differentiation and proliferation (9–12).

Numerous risk factors—both modifiable and non-modifiable—also contribute to CRC carcinogenesis. A family history of CRC significantly increases risk; first-degree relatives of CRC patients have a threefold increased risk, with a relative risk of 1.8 (1, 13). Genetic syndromes such as familial adenomatous polyposis (FAP), Peutz–Jeghers syndrome, and Lynch syndrome also elevate risk (1). Individuals with inflammatory bowel disease (IBD) face a relative risk of 2.93 (13). Continuing with non-modifiable risk factors, men have a higher incidence of CRC compared to women, with a ratio of 1.3:1 (1). Dietary and lifestyle factors also play a major role. Diets high in fat and low in fiber may increase exposure to intestinal carcinogens via bile metabolism (1). Smoking and alcohol are also associated with increased risk. For example, 30 pack-years of smoking raises the relative risk to 1.26, as does consuming 20 alcoholic drinks per week (13). While there are many factors which increased an individual’s risk for CRC, there are also protective actions one can take. Regular physical activity, a diet rich in fruits and vegetables, and the use of aspirin or NSAIDs have been shown to reduce CRC risk (13).

Given the significant impact of CRC, screening is a critical component of preventative care. The U.S. Preventive Services Task Force currently recommends (Grade A) screening for adults aged 50–75 and (Grade B) screening for adults aged 45–49 (14). Screening options include annual stool-based tests followed by colonoscopy if positive, or colonoscopy every 10 years (14). Over the past 25 years, these guidelines have evolved. In June 2002, screening was recommended for all individuals aged 50 and older with no recommendation for appropriate age to phase out screening (14). By 2016, guidelines added this recommendation and supported screening adults ages 50-75 (14). In 2021, the USPSTF expanded screening to include adults starting at age 45 due to emerging data (14). The rationale for lowering the screening age is multifactorial. Firstly, development of technology and research has allowed for a better understanding of the disease and its risk factors. Secondly, epidemiologic data has revealed a sharp increase in CRC among younger adults (15). In 2019, 20% of CRC diagnoses occurred in individuals under 55—nearly double the proportion in 1995—despite this age group shrinking as a share of the overall population (15). Of these, only 10–20% of early-onset cases are linked to inherited syndromes such as Lynch (16). Mortality trends are also concerning: CRC deaths among individuals younger than 50 rose by 1% annually from 2011 to 2020, while death rates declined in older age groups (15). The generational shift in CRC incidence suggests significant environmental and lifestyle influences (16).

Various theories have been proposed to better understand the underlying factors driving this trend. While modern society offers many advantages, it also comes with often-overlooked trade-offs. The pursuit of convenience has increased human exposure to chemicals through pollution, pesticides, and plastics (16). Researchers are increasingly concerned that contact with these substances may lead to genetic mutations, potentially predisposing individuals to malignancy (16). However, chemical exposure is not the only concern. Modern lifestyles are also characterized by lower fiber intake, higher consumption of processed foods, and reduced physical activity—all factors associated with increased cancer risk. Obesity, which has become more prevalent, is linked to chronic inflammation, oxidative stress, hormonal imbalances, and growth factor dysregulation, each of which can promote tumorigenesis (17). Another growing area of interest is the gut microbiome. This complex ecosystem is shaped by diet, environment, medications, and lifestyle. Disruptions to the gut microbiota can activate inflammatory pathways, ultimately compromising the body’s natural defense mechanisms and potentially increasing the risk of CRC (18).

Given the rising incidence of CRC, particularly among younger populations, the importance of early detection cannot be overstated. By identifying precancerous polyps or early-stage tumors, screening not only reduces mortality but also offers an opportunity for prevention. As our understanding of the environmental, genetic, and lifestyle factors influencing CRC deepens, so too must our commitment to proactive healthcare. Promoting awareness, encouraging routine screening, and supporting accessible care are vital steps in reducing the burden of this disease. While national efforts are often viewed as the primary means to achieve these goals—a narrow regional, or community-centered, approach may support improved outcomes as opposed to a one-size-fits-all approach as it is tailored to the specific needs of those within it. With this study we aim to assess the presentation of a small proportion of CRC patients in the greater Toledo area to both identify factors that may correlate to CRC incidence in younger age groups, and to identify primary risk factors of this population in order to direct public health initiatives based on our findings.

## Methods

### Data collection

Patient charts from the Department of Hematology and Oncology at Toledo Clinic, a private, multi-center outpatient clinic which serves patients from multiple counties in the Northwest Ohio region (including Lucas, Wood, Fulton, Henry, Ottawa, and Sandusky), were accessed remotely via OncoEMR for the timeframe of January 1^st^, 2018 through July 1^st^, 2023. Patients were qualified to be enrolled into the study if they had a primary CRC that was staged between Stage 0 and Stage IVC, and if they were diagnosed within this timeframe. New patients to the clinic with CRC that had a diagnosis made prior to, or after, this window of time were excluded from the study. Patients did not need to be originally diagnosed at Toledo Clinic to qualify for enrollment. In total, there were 230 patients who qualified for further evaluation in this study. The chart for each enrolled patient was reviewed thoroughly, and all relevant demographic information and disease-specific data was recorded. The collected demographic data for each patient included: race, sex, date of birth, veteran status, known predisposing environmental risk factors, and BMI. A patient’s BMI was determined by the height and weight they were measured at during their initial appointment, with a BMI of 25.0-29.9 being classified as overweight, and a BMI ≥ 30 being classified as obese. Patient risk factors such as type-2 diabetes mellitus (T2DM) and dyslipidemia were noted as previously diagnosed chronic conditions by the patient’s primary care physician, with T2DM being diagnosed from a hemoglobin A1C of ≥ 6.5% or a fasting serum glucose of ≥ 126, and with dyslipidemia being diagnosed from a total cholesterol ≥ 200, LDL ≥ 130, HDL < 40 in men and < 50 in women, and triglycerides ≥ 145. The collected disease-specific data included: the date of diagnosis, stage at diagnosis, site of the primary tumor, patient genetic data, tumor molecular data from Tempus testing of solid tumor biopsy samples, tumor programmed death-ligand 1 (PD-L1) expression levels, and patient outcome (listed as either surveillance/stable disease, lost to follow-up/care transitioned, disease progression/disease recurrence, and deceased). For patient outcomes, surveillance/stable disease was defined as patients who successfully completed primary treatment and are undergoing routine follow-up based on current guideline recommendations without clinical, radiographic, or pathological evidence of disease recurrence or progression. Lost to follow-up/care transitioned was defined as patients who unexpectedly failed to attend scheduled follow-up visits for ≥ 6 months without indication of returning and additional clinical data, or patients who officially had their care transferred to another institution for which additional patient records were inaccessible. Disease progression/disease recurrence was defined as worsening of disease during or after primary treatment noted through clinical, radiographic, or pathological evidence, or the reappearance of disease confirmed with clinical, radiographic, or pathological evidence after previously having no evidence of disease. Deceased was defined as officially documented death from any cause while actively receiving evaluation or treatment for their disease. All patient-specific identifiers, specifically the patient’s medical record number, were removed after the collection of patient data was completed prior to data analysis was performed.

### Statistical analysis

Patients were stratified by age at diagnosis into decade-based groups (30–39, 40–49, etc.), with further comparison between younger (30–49 years) and older (50+ years) cohorts. Continuous variables were summarized as means ± standard deviation or medians with interquartile ranges, and categorical variables as frequencies and percentages. Genetic testing coverage across age groups was compared using Chi-square tests, conducted in Microsoft Excel and verified using an online calculator (https://www.socscistatistics.com). Mutation prevalence was calculated for each gene among patients with available testing results, using only those tested for a given gene as the denominator. Risk factor associations were assessed with 2×2 contingency tables for each clinical risk factor-gene pair, using Chi-square or Fisher’s Exact Tests depending on expected cell sizes (https://astatsa.com/FisherTest/). Associations between mutation status and clinical outcomes (e.g., stage at diagnosis, primary tumor site) were analyzed per gene. Code generation, data visualization, and organizational assistance were provided by Claude Opus 4 (Anthropic, 2025), and Microsoft Excel with all calculations verified by a human author (LR). Statistical tests included Chi-square for comparisons with expected cell counts ≥ 5 and Fisher’s Exact for those < 5. Odds ratios (OR) with 95% confidence intervals were reported for significant associations. Given the exploratory nature and volume of comparisons (n = 42), unadjusted p-values are reported, with p < 0.05 considered nominally significant. Patients with missing data were excluded from relevant analyses, and final sample sizes are reported per analysis. All statistical work was performed in Python 3.x using pandas (v1.3.0), SciPy (v1.7.0), and Google Collab for visualization, with figures and tables also generated in Microsoft Excel.

## Results

### Evaluation of patient demographics and presentation

**Figure 1:**
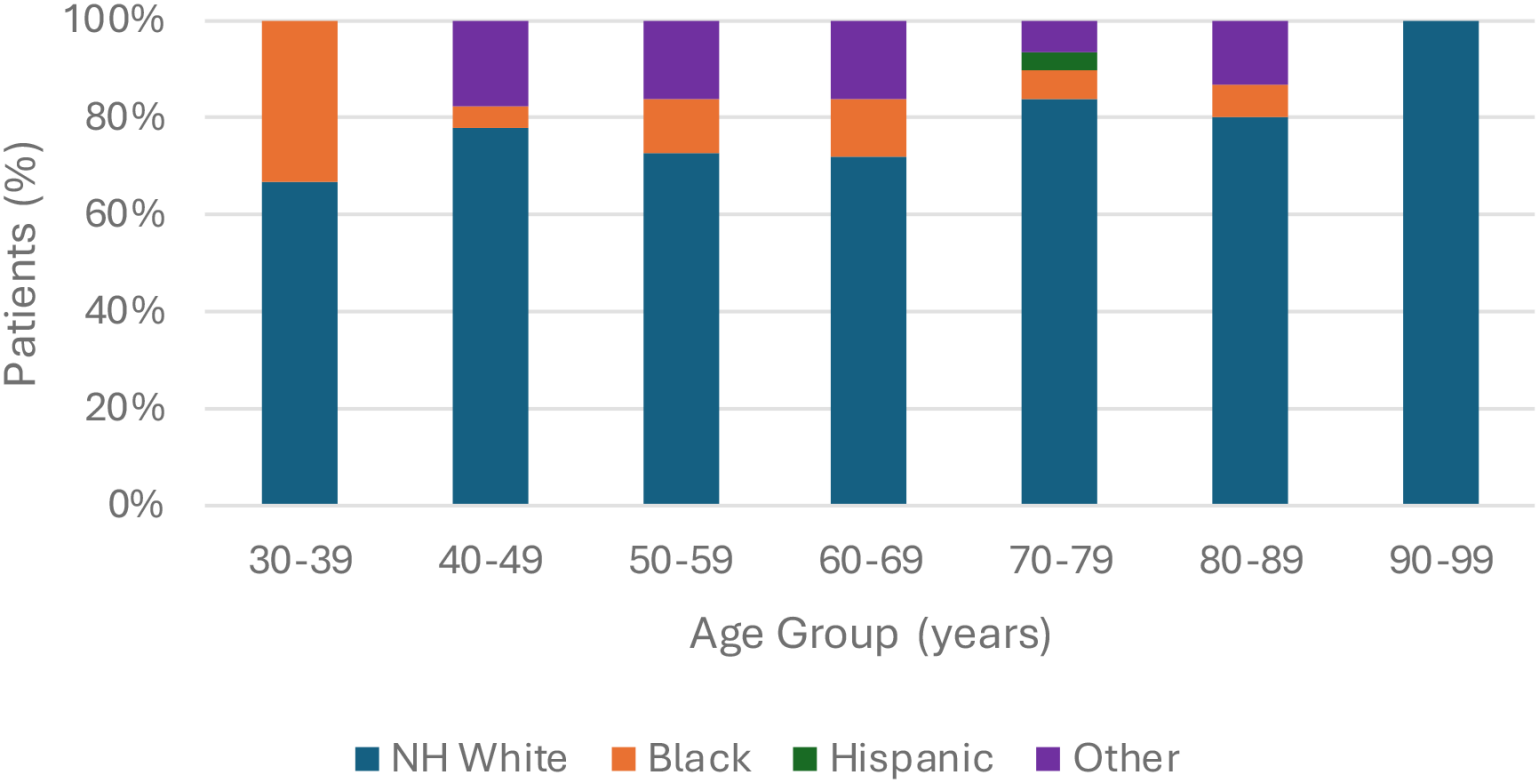
Race distribution by age group.

**Figure 2:**
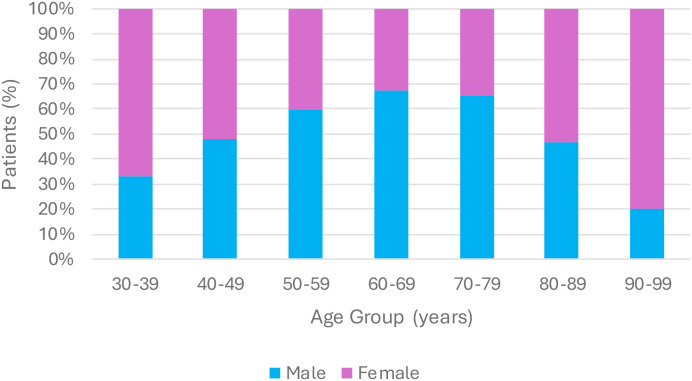
Sex distribution by age group.

In our pool of qualifying patients, we had 6 patients aged 30-39, 23 patients aged 40-49, 37 patients aged 50-59, 68 patients aged 60-69, 61 patients aged 70-79, 30 patients aged 80-89, and 5 patients aged 90-99. No patients were identified who were 20–29 years of age. Cumulatively, we had 178 patients (77.4%) who identified as non-Hispanic White, 21 patients (9.1%) who identified as Black, and 31 patients (13.5%) who identified as either Hispanic or Other (including Asian, Pacific Islander, and Multiracial), and we had 136 patients who identified as male (59.1%) and 94 patients who identified as female (40.9%). These groups have been stratified by age group in **Figure 1** and **Figure 2**, respectively.

**Figure 3:**
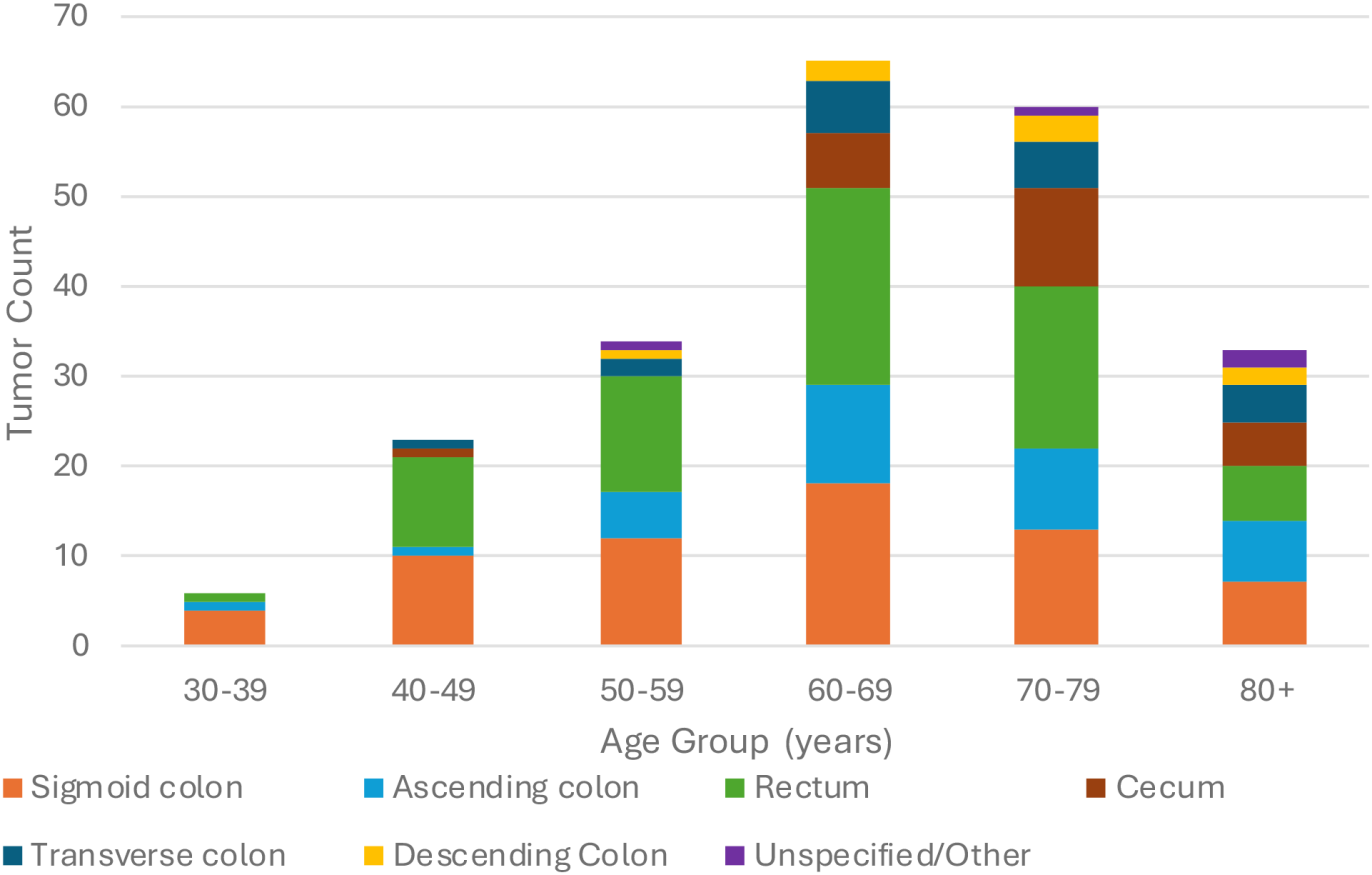
Primary tumor site by age group.

In our population, 70 patients had a primary cancer of the rectum, 71 patients had a primary cancer of the sigmoid colon, 8 patients had a primary cancer of the descending colon, 17 patients had a primary cancer of the transverse colon, 32 patients had a primary cancer of the ascending colon, 23 patients had a primary cancer of the cecum, 3 patients had two primary cancer sites (rectum and ascending colon; transverse colon and descending colon; sigmoid colon and ascending colon), 1 patient had three primary cancer sites (cecum, ascending colon, and rectum), and 5 patients had pan-colonic or otherwise unspecified primary cancer sites. Primary tumor locations have been stratified by age group in **Figure 3**.

**Figure 4:**
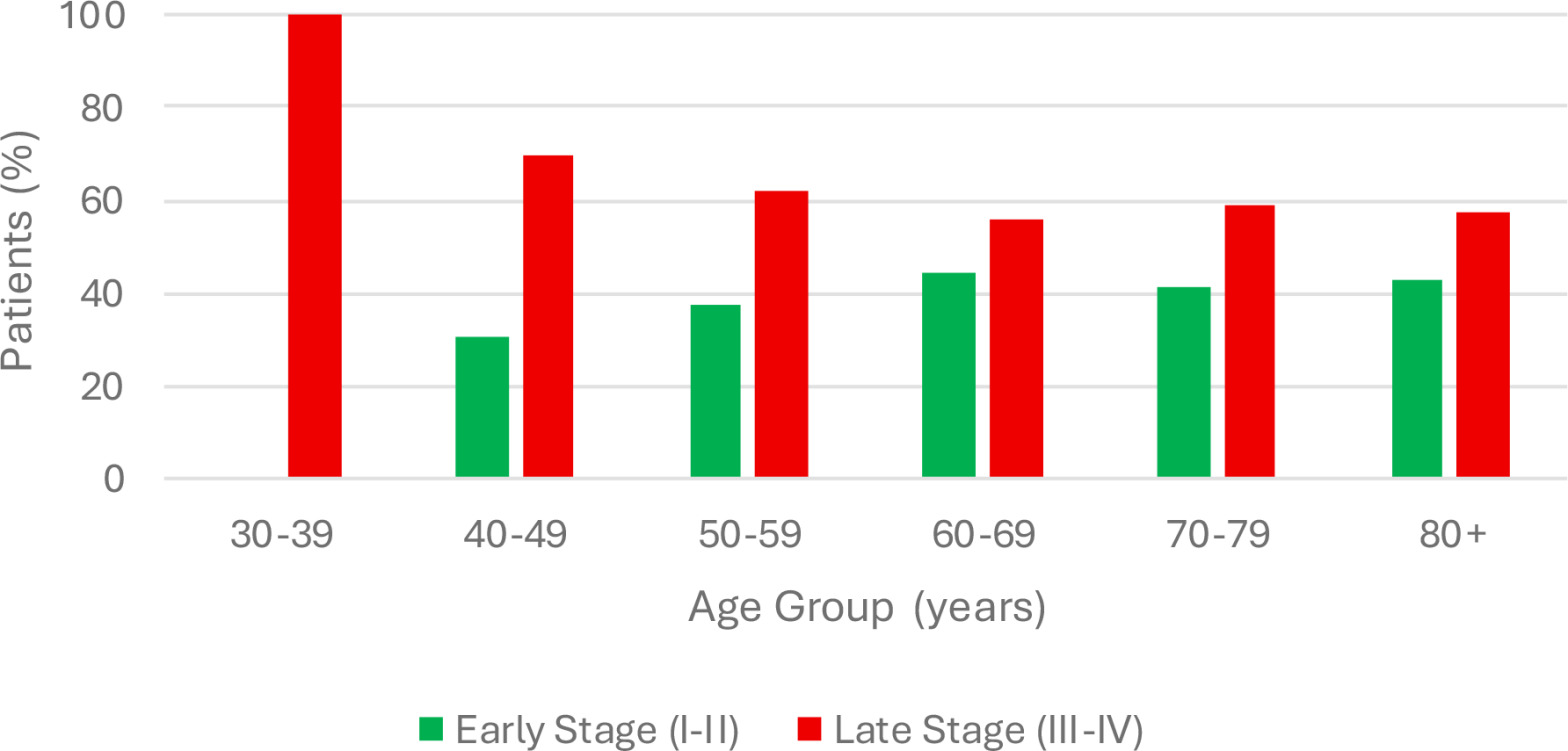
Stage at diagnosis distribution by age group.

**Figure 5:**
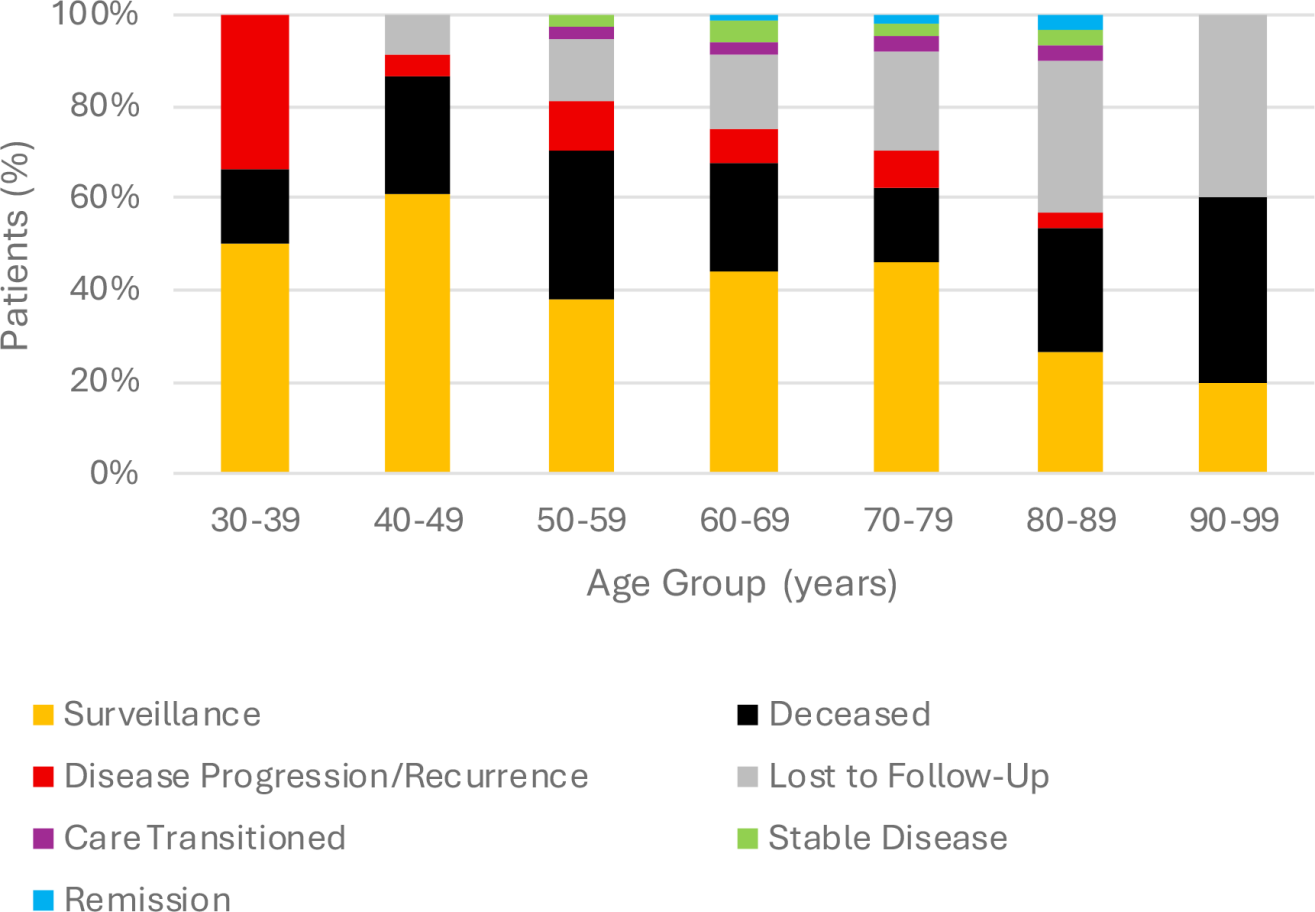
Outcome distribution by age group.

Additionally, 32 patients presented with stage I disease, 51 patients presented with stage II disease, 81 patients presented with stage III disease, and 63 patients presented with stage IV disease, and 3 patients had unconfirmed staging. Disease stage at diagnosis has been stratified by age group in **Figure 4**. Lastly, after undergoing treatment, we had 108 patients ultimately have disease control (either as remission, surveillance, and stable disease), 18 patients with active disease, 49 patients who were lost to follow-up or transferred their care to another facility, and 55 patients who ultimately passed away due to their disease. These outcomes have been stratified by age groups in **Figure 5**. As a control to assess the quality of our patient pool, a chi-square test was performed comparing the stage at diagnosis to patient outcome. As expected, this showed that patients with late-stage disease (stage III and stage IV) were found to have significantly worse outcomes than those with early-stage disease (stage I and stage II), as χ² = 41.81, p < 0.00001, OR 2.70 (95% CI 1.56-4.69).

**Figure 6:**
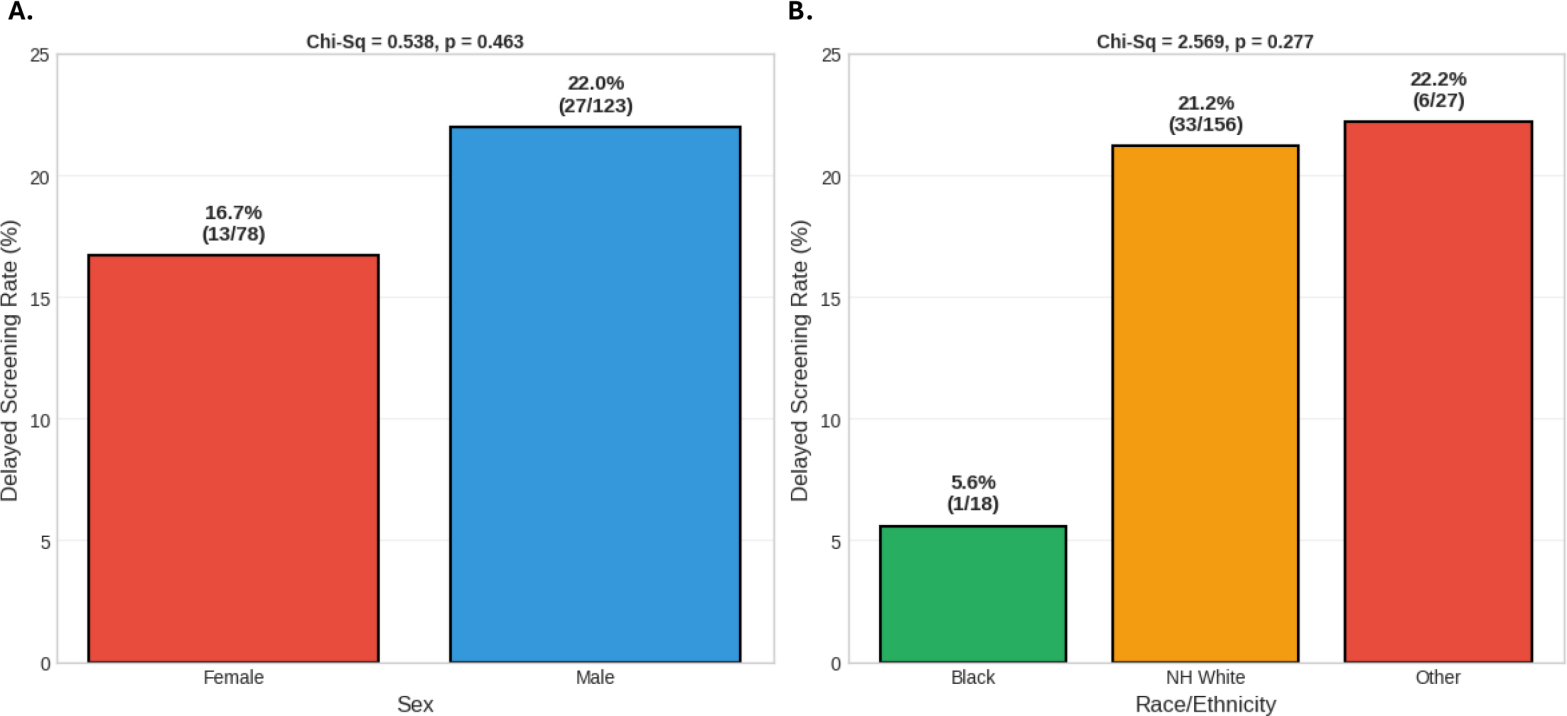
Colonoscopy screening disparities in colorectal cancer patients ≥ 50 years old. **(A)** Delayed screening rate by sex, **(B)** Delayed screening rate by race.

Lastly, we examined the rate at which screening colonoscopies were delayed amongst our patient population and compared these findings across both patient race and sex. In our study, 22.0% of patients who identified as male reported a delay in obtaining their screening colonoscopy, whereas 16.7% of patients who identified as female reported a delay in obtaining their screening colonoscopy. The difference in proportions was 5.3%, corresponding to an odds ratio (OR) of 1.38 (95% CI 0.70–2.71). A chi-square test with Yates correction showed that this was not a significant difference, as χ² = 0.54, p > 0.05 (**Figure 6A**). Furthermore, 21.2% of non-Hispanic White patients reported a delay in obtaining their screening colonoscopy, whereas 5.6% of Black and 22.2% of patients who identified as Other reported a delay in obtaining their screening colonoscopy, respectively. A chi-square test showed that this was also not a significant difference between these groups, as χ² = 2.57, p > 0.05 (**Figure 6B**).

### The effect of patient demographics on disease stage at diagnosis and treatment outcome

The stage of disease at the time of diagnosis was compared between racial groups, specifically assessing the frequency that patients presented with late-stage disease (stage III and stage IV). In our group of patients across all age groups, 62.3% of non-Hispanic White patients presented with late-stage disease, 38.1% of Black patients presented with late-stage disease, and 71% of patients who identified as Other presented with late-stage disease. Our non-White, non-Black patients were found to have significantly more disease burden at the time of diagnosis compared to both non-Hispanic White and Black patients, as a chi-square test showed χ² = 10.59, p = 0.013, OR 0.37 (95% CI 0.15-0.95) (**Table 1**).

**Table 1:** Disease stage across patient demographics.

Demographic	Early-stage	Late-stage	Total	Late-stage rate	P-value	OR (95% CI)
Presentation by patient race						
Black	13 (61.9%)	8 (38.1%)	21 (9.3%)	38.10%		
Non-Hispanic White	66 (37.7%)	109 (62.3%)	175 (77.1%)	62.30%		
Other	9 (29.0%)	22 (71.0%)	31 (12.8%)	71.00%		
**Total**	**88 (38.8%)**	**139 (61.2%)**	**227 (100%)**	**61.20%**	**0.013**	**0.37 (0.15-0.95)**
Presentation by patient sex						
Female	32 (34.4%)	61 (65.6%)	93 (41.0%)	65.60%		
Male	56 (41.8%)	78 (58.2%)	134 (59.0%)	58.20%		
**Total**	**88 (38.8%)**	**139 (61.2%)**	**227 (100%)**	**61.20%**	**0.262**	**1.37 (0.79–2.37)**

Similarly, the stage of disease at the time of diagnosis was compared between male and female patients, specifically assessing the frequency that patients presented with late-stage disease. In our group of patients across all age groups, 58.2% of patients who identified as male presented with late-stage disease, whereas 65.6% of patients who identified as female presented with late-stage disease. A chi-square test comparing disease stage and patient sex showed no significant difference, as χ² = 1.26, p > 0.05, OR 1.37 (95% CI 0.79-2.37) (**Table 1**).

Outcomes of patients were then compared between patient race and patient sex to identify if disparities in patient care existed during treatment. Of non-Hispanic White patients across all age groups, 47.8% had disease control, 6.2% had disease progression, 25.3% passed away, and 20.8% were either lost to follow-up or had their care transitioned to another facility. Of Black patients across all age groups, 38.1% had disease control, 19.1% had disease progression, 9.5% passed away, and 33.3% were either lost to follow-up or had their care transitioned to another facility. Of patients who identified as Other across all age groups, 51.7% had disease control, 10.3% had disease progression, 27.6% passed away, and 17.2% were either lost to follow-up or had their care transitioned to another facility. A chi-square test comparing the outcomes of these groups did not show a significant difference in outcomes, as χ² = 8.36, p > 0.05 (**Table 2**). Broader comparison of non-Hispanic White patients and non-White patients also failed to show a significant difference in outcomes using a chi-square test, as χ² = 3.55, p > 0.05.

**Table 2:** Patient outcomes across patient demographics.

Demographic	Deceased	Active disease/disease progression/recurrence	Disease control*	Other/Lost to follow-up	p-value OR (95% CI)
OUTCOME BY PATIENT RACE					
Black	2 (9.5%)	4 (19.1%)	8 (38.1%)	7 (33.3%)	0.013
Non-Hispanic White	45 (25.3%)	11 (6.2%)	85 (47.8%)	37 (20.8%)	
Other	8 (27.6%)	3 (10.3%)	15 (51.7%)	5 (17.2%)	
OUTCOME BY PATIENT SEX					
Female	18 (19.4%)	7 (7.5%)	48 (51.6%)	20 (21.5%)	0.379 1.32 (0.77-2.24)
Male	36 (26.9%)	11 (8.2%)	60 (44.8%)	27 (20.1%)	

*Includes patients with surveillance, stable disease, and remission status.

Furthermore, of patients who identified as male across all age groups, 44.8% had disease control, 8.2% had disease progression, 26.9% passed away, and 20.1% were either lost to follow-up or had their care transitioned to another facility. For patients who identified as female across all age groups, 51.6% had disease control, 7.5% had disease progression, 19.4% passed away, and 21.5% were either lost to follow-up or had their care transitioned to another facility. These findings were found to also to lack statistical significance using a chi-square test, as χ² = 1.92, p > 0.05 (**Table 2**).

### The effect of known colorectal cancer risk factors on disease stage at diagnosis

We further analyzed our patient population by comparing the presence of comorbid risk factors for CRC—including type-2 diabetes, dyslipidemia, elevated body mass index (BMI), tobacco use, and heavy alcohol use classified as ≥ 3 standard drinks per day —with the stage of disease at the time of diagnosis. Our goal was to assess if any particular risk factor was correlated with a higher likelihood of advanced disease at diagnosis than others, and if the presence of multiple risk factors predisposed patients to more-advanced disease than those with fewer risk factors. An evaluation of the frequency of these risk factors stratified by age group can be seen in **Table 3**.

**Table 3:** Risk factor prevalence across patient age groups.

Age group	n (%)	Late-Stage %	Overweight/obese %	Tobacco use %	Heavy Alcohol Use %	T2DM %	Dyslipidemia %
30-39	6 (2.6)	100	83.3	16.7	0	16.7	16.7
40-49	23 (10.0)	69.6	82.6	52.2	4.3	17.4	8.7
50-59	37 (16.1)	62.2	75.7	43.2	10.8	10.8	18.9
60-69	68 (29.6)	55.8	73.5	64.7	17.6	29.4	42.6
70-79	61 (26.5)	59	80.3	60.7	9.8	31.1	75.4
80+	35 (15.2)	57.1	60	37.1	0	37.1	48.6

Late-stage is defined as Stage III-IV at the time of diagnosis. Heavy alcohol use is defined as 3+ drinks daily.

We began by assessing metabolic disorders, specifically diabetes and dyslipidemia. In total, across all age groups, 5 patients had diabetes only (4 early-stage, 1 late-stage), 56 patients had dyslipidemia only (36 early-stage, 20 late-stage), 44 patients had both diabetes and dyslipidemia (32 early-stage, 12 late-stage), and 122 patients had neither disorder (16 early-stage, 106 late-stage). Conducting a chi-square test comparing patients with either diabetes, dyslipidemia, or both conditions showed no significant difference in stage of disease at the time of diagnosis, as χ² = 1.13, p > 0.05. Oddly, a chi-square test showed that patients without either metabolic disorder presented with significantly more-advanced disease at the time of diagnosis compared to those with one, or both, metabolic disorders, as χ² = 74.14, p < 0.00001. A similarly significant result was observed when comparing the group of patients with both metabolic disorders and the group of patients without either condition, as χ² = 55.91, p < 0.00001. Despite being statistically significant, we believe that this is an artifact of follow-up intensity in our patient cohort rather than a true biological association.

Additionally, in our patient population, 85 patients were overweight (36 early-stage, 49 late-stage) and 85 were obese (30 early-stage, 55 late-stage), 121 patients had a history of tobacco use (55 early-stage, 66 late-stage), 22 patients reported heavy alcohol use (10 early-stage, 12 late-stage), 18 patients who had tobacco use history and reported heavy alcohol use (10 early-stage, 8 late-stage), 72 patients who were overweight/obese with tobacco use history (33 early-stage, 39 late-stage), 3 patients who were overweight/obese and reported heavy alcohol use (0 early-stage, 3 late-stage), and 13 patients who were overweight/obese with tobacco use history and reported heavy alcohol use (7 early-stage, 6 late-stage). A chi-square test comparing the difference in the stage of disease at the time of diagnosis in overweight and obese patients showed no significant difference (χ² = 0.89, p = 0.345), so we combined these patients into one group during further analyses. Furthermore, a two-sided Fisher’s Exact Test comparing patients from each of the aforementioned risk factor groups showed no significant difference in the stage of disease at the time of diagnosis between them, as p > 0.05 (**Table 4**).

**Table 4:** Patient outcomes by risk factor.

Risk factor category	Total n	Early-stage n (%)	Late-stage n (%)	p-value OR (95% CI)
Metabolic disorders				
Diabetes only	5	4 (80.0)	1 (20.0)	p = 0.567 0.45 (0.05-4.31)
Dyslipidemia only	56	36 (64.3)	20 (35.7)	
Both diabetes and dyslipidemia	44	32 (72.7)	12 (27.3)	
Substance use/Lifestyle factors				
Overweight/Obese	170	67 (39.4)	103 (60.6)	p = 0.528
History of Tobacco Use	121	55 (45.5)	66 (54.5)	
Heavy Alcohol Use (≥3 drinks/day)	22	10 (45.5)	12 (54.5)	
Tobacco + Alcohol	18	10 (55.6)	8 (44.4)	
Overweight/Obese + Tobacco	72	33 (45.8)	39 (54.2)	
Overweight/Obese + Alcohol	3	0 (0)	3 (100)	
Overweight/Obese + Tobacco + Alcohol	13	7 (53.8)	6 (46.2)	

### Disease severity at the time of diagnosis across age groups

Next, we wanted to determine if there was a significant difference the stage of disease at the time of diagnosis across age groups, with an emphasis on comparing younger patients (30–49 years of age) to older patients (≥ 50 years of age). Stratifying patients by age group, 100% (6/6) of patients aged 30–39 presented with late-stage disease, 69.6% (16/23) of patients aged 40–49 presented with late-stage disease, 63.9% (23/36; 1 patient un-staged) of patients aged 50–59 presented with late-stage disease, 56.7% (38/67; 1 patient un-staged) of patients aged 60–69 presented with late-stage disease, 60.0% (36/60; 1 patient un-staged) of patients aged 70–79 presented with late-stage disease, 53.3% (16/30) of patients aged 80–89 presented with late-stage disease, and 80% (4/5) of patients aged 90–99 presented with late-stage disease. By extension, 75.9% (22/29) of patients aged 30–49 presented with late-stage disease, and 59.1% (117/198) of patients aged ≥ 50 years of age presented with late-stage disease. A two-sided Fisher’s Exact Test comparing disease severity across all age groups showed there was no significant difference, as p > 0.05 (**Table 5**). When comparing disease severity among patients aged 30–49 against patients aged ≥ 50, a chi-square test also did not show a significant difference with χ² = 2.99 (p = 0.083; OR 2.18; 95% CI 0.89-5.33) (**Table 5**).

**Table 5:** Statistical comparisons of stage at diagnosis by age groups.

Test	p-value, OR (95% CI)	Interpretation
Analysis 1: Fisher’s Exact Test between all age groups	p = 0.324	Not significant
Analysis 2: Chi-square test of patients 30–49 vs 50+	p = 0.083 2.18 (0.89-5.33)	Not significant

Statistical significance defined as p < 0.05. Despite lack of statistical significance, the clinical finding of 100% late-stage disease in patients aged 30–39 is intriguing but should only be considered hypothesis-generating given the small patient count in this age group (n = 6).

### Evaluation of tumor molecular alterations

Finally, our analysis of our patient population then extended to the presence or absence of tumor molecular alterations. Of our 230 patients, 145 (63.0%) had documented tumor molecular testing performed. In total, 83.3% of patients aged 30–39 had tumor molecular testing performed, 95.7% of patients aged 40–49 had tumor molecular testing performed, 67.6% of patients aged 50–59 had tumor molecular testing performed, 55.9% of patients aged 60–69 had tumor molecular testing performed, 59.0% of patients aged 70–79 had tumor molecular testing performed, 56.7% of patients aged 80–89 had tumor molecular testing performed, and 40.0% of patients aged 90–99 had tumor molecular testing performed. We then identified the mutational status of major genes associated with CRC—including NRAS, KRAS, BRAF, TP53, and APC—in addition to the status of mismatch repair (MMR) genes, and microsatellite stability for each patient who had tumor molecular testing performed. A comparison of tumor PD-L1 expression levels between our patients was also planned, however there was an inadequate number of patients with this testing documented, and no analysis could be performed. Across all age groups, 48 patients were found to have tumors with APC mutations, 43 patients were found to have tumors with TP53 mutations (with two patients having tumors that had two TP53 variants detected). 33 patients were found to have tumors with KRAS mutations (with one patient having a tumor with two mutant KRAS variants detected), 11 were found to have tumors with BRAF mutations (with one patient having a tumor with two mutant BRAF variants detected), and 1 patient was found to have an NRAS mutation. Unfortunately, we did not have an adequate pool of patients with tumors that had MMR deficiencies, so analysis of these genes was excluded.

**Figure 7:**
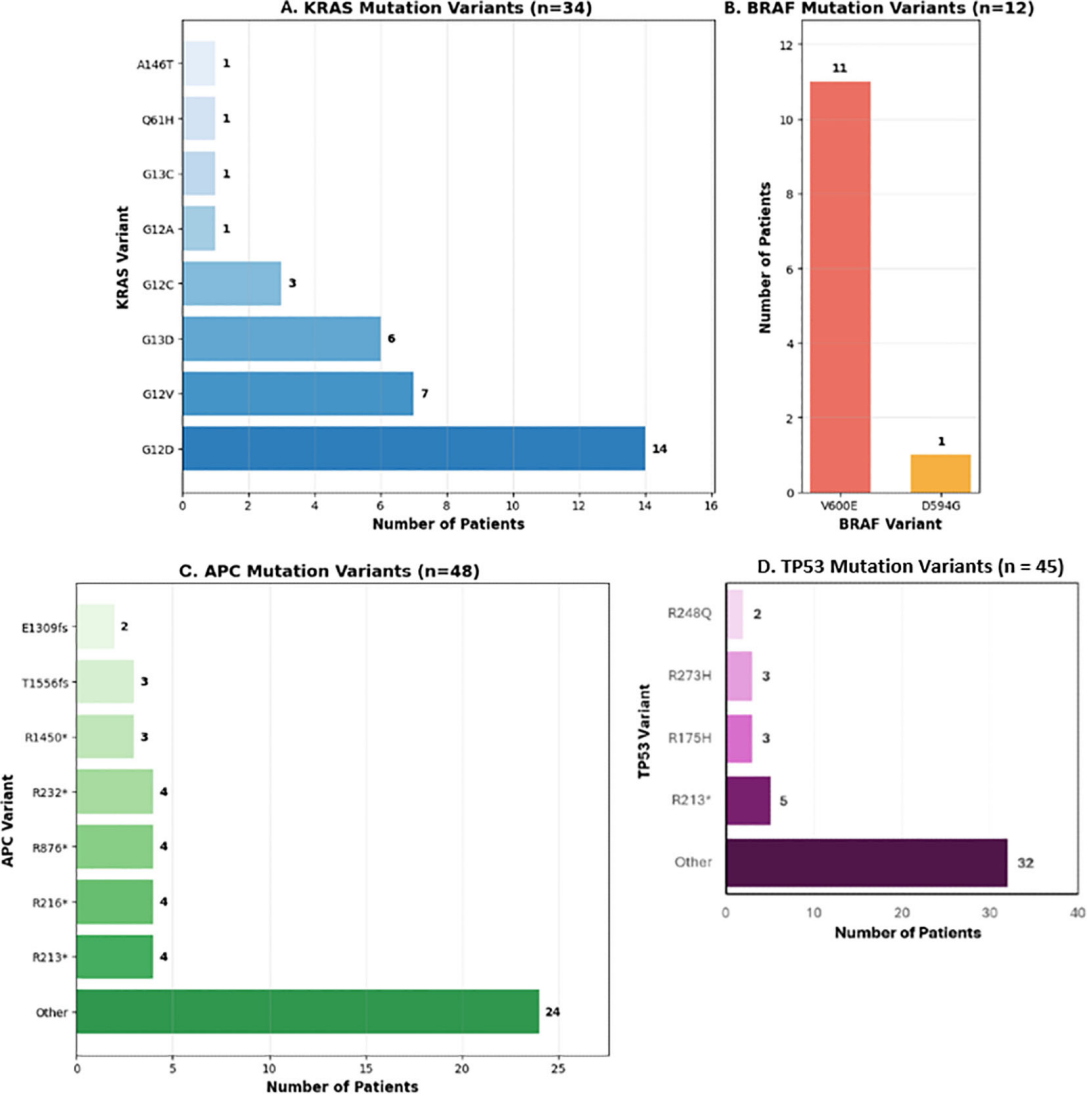
Distribution of specific tumor molecular variants in colorectal cancer patients.

In our patient population across all age groups, KRAS G12D was the most represented variant (42.4%) in patients with tumors that had mutated KRAS, and BRAF V600E was the most represented variant (100%) in patients with tumors that had mutated BRAF. However, patients with tumors that had mutated APC did not have a clearly defined variant, as 50.0% of patients had unique mutations. Despite this, nonsense mutations at R213, R216, R232, and R876 were the most common variants, with each variant being present in 8.3% of patients that had tumors with an APC mutation. Similarly, the majority (74.4%) of patients with tumors that had a TP53 mutation did not have a clearly defined variant, however a nonsense mutation at R213 was the most common variant (11.6%). These findings have been outlined in **Figure 7**.

To begin, we compared the stage of disease at the time of diagnosis with the presence or absence of NRAS, KRAS, BRAF, and APC variants in tumor samples from our patients. Chi-square tests comparing patient sex to the presence of tumor-specific KRAS mutational status, BRAF mutational status, and TP53 mutational status across all age groups showed no significant difference, with tumor-specific KRAS mutational status having χ² = 0.15 (OR 1.25; p = 0.70; 95% CI 0.58-2.69), tumor-specific BRAF mutational status having χ² = 0.00 (OR 0.83; p = 1.00; 95% CI 0.24-2.79), and tumor-specific TP53 mutational status having χ² = 2.01 (OR 1.78; p = 0.16; 95% CI 0.87-3.62). However, a chi-square test comparing patient sex to tumor-specific APC mutational status across all age groups suggested that male sex is associated with a higher prevalence of tumors with oncogenic APC variants compared to tumors found in patients of female sex, as χ² = 4.15 (OR 2.18; p = 0.032; 95% CI 1.08-4.39). These results remained consistent after adjusting for patient age and race, with tumor-specific KRAS mutational status having an adjusted OR 1.39 (p = 0.70; 95% CI 0.58-2.69), tumor-specific BRAF mutational status having an adjusted OR 0.93 (p = 0.91; 95% CI 0.27-3.19), tumor-specific APC mutational status having an adjusted OR 2.24 (p = 0.03; 95% CI 1.08-4.63), and tumor-specific TP53 mutational status having an adjusted OR 1.96 (p = 0.07; 95% CI 0.93-4.15).

**Figure 8:**
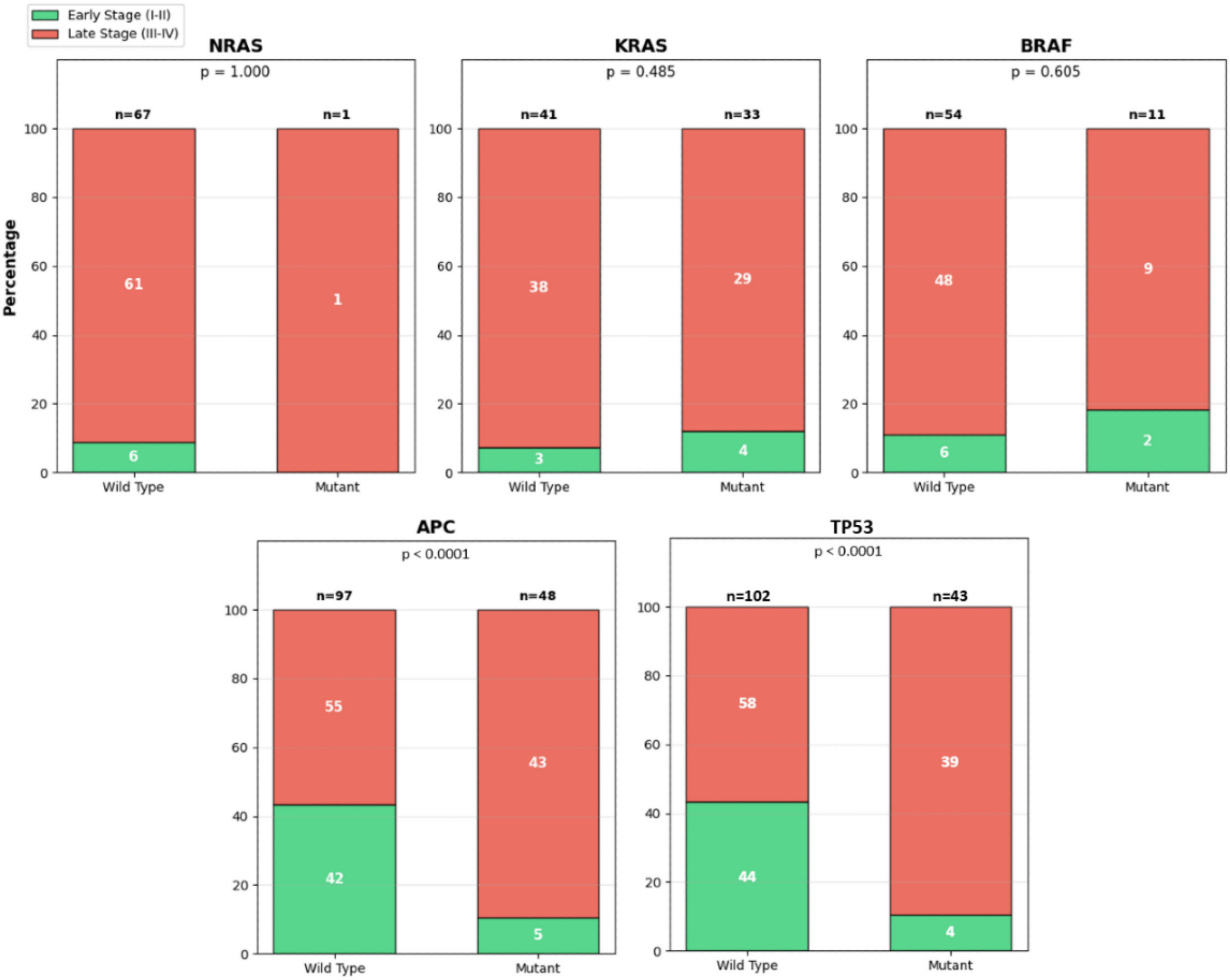
Stage at diagnosis by tumor molecular alteration status.

Next, a Fisher’s Exact Test comparing disease severity to tumor-specific NRAS mutational status across all age groups showed there was no significant difference, as p = 1.000, which was largely in part due to the presence of only 1 patient with a tumor-specific NRAS mutation (**Figure 8**). A chi-square test comparing disease severity to tumor-specific KRAS mutational status across all age groups showed no significant difference (χ² = 0.49; p = 0.485; OR 0.57; 95% CI 0.12-2.76), and another chi-square test comparing disease severity to tumor-specific BRAF mutational status across all age groups also showed no significant difference (χ² = 0.42; p = 0.605; OR 1.15; 95% CI 0.32-4.10) (**Figure 8**). However, chi-square tests comparing disease severity to tumor-specific APC mutational status and tumor-specific TP53 mutational status across all age groups showed a significant difference with both genes, as χ² = 15.85 (p < 0.0001), OR 3.57 (95% CI 1.60-7.97), and χ² = 15.637 (p < 0.0001), OR 3.36 (95% CI 1.46-7.71), respectively. These results remain consistent even after adjusting for patient age, race, sex. primary tumor site, and risk factors, with comparisons between disease severity and tumor-specific KRAS mutational status having an adjusted OR 0.32 (p = 0.32; 95% CI 0.03-3.02), tumor-specific BRAF mutational status having an adjusted OR 0.37 (p = 0.46; 95% CI 0.03-5.14), tumor-specific APC mutational status having an adjusted OR 9.51 (p < 0.0001; 95% CI 3.36-26.90), and tumor-specific TP53 mutational status having an adjusted OR 8.36 (p < 0.001; 95% CI 2.78-25.37) Thus supporting the notion that advanced disease is associated with a higher prevalence of oncogenic APC and TP53 variants in our patients (**Figure 8**).

### Comparing tumor molecular alterations across age groups

**Figure 9:**
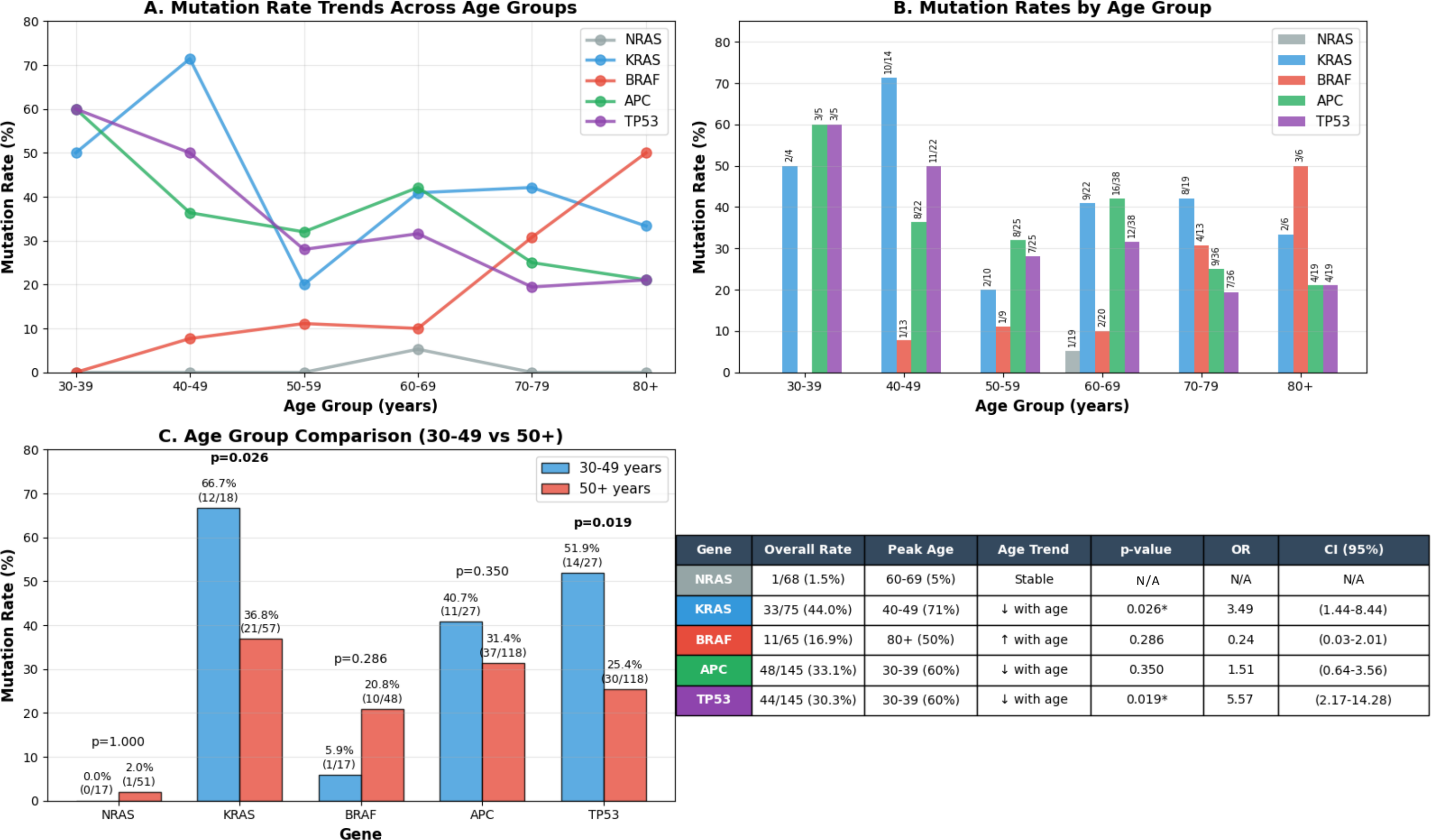
Comprehensive analysis of tumor molecular alteration patterns by age group.

Next, we evaluated the presence of tumor-specific NRAS, KRAS, BRAF, and APC mutations across age groups, specifically comparing patients who were 30–49 years of age and those who were ≥ 50 years of age. In our patients who had an assessment of tumor molecular alterations performed, 5.9% of patients aged 30–49 and 20.8% of patients aged ≥ 50 had a tumor-specific BRAF mutation, 40.7% of patients aged 30–49 and 31.4% of patients aged ≥ 50 had a tumor-specific APC mutation, and 66.7% of patients aged 30–49 and 36.8% of patients aged ≥ 50 had a tumor-specific KRAS mutation. Unfortunately, a tumor-specific NRAS mutation was only identified in one patient who was ≥ 50 years of age, thus no analysis could be performed. A chi-square test comparing the prevalence of tumor-specific pathologic variants of BRAF and APC between patients who were 30–49 years of age and those who were ≥ 50 years of age showed no significant difference, as χ² = 1.99, p = 0.158, OR 0.30 (95% CI 0.07-1.34); and χ² = 0.87, p = 0.350, OR 1.51 (95% CI 0.64-3.56) respectively (**Figure 9**). However, a chi-square test identified that patients aged 30–49 were significantly more likely to present with tumor-specific KRAS mutations (χ² = 4.94; p = 0.026; OR 3.49; 95% CI 1.44-8.44), and tumor-specific TP53 mutations (χ² = 5.43; p = 0.019; OR 4.55; 95% CI 1.99-10.42) (**Figure 9**). These results remain significant when adjusting for race and sex, with the presence of tumor-specific KRAS mutations in patients aged 30–49 having an adjusted OR of 4.11 (p = 0.019; 95% CI 1.26-13.40) and tumor-specific TP53 mutations in patients aged 30–49 having an adjusted OR of 5.30 (p = 0.00018; 95% CI 2.22-12.69).

### The association of colorectal cancer risk factors and oncogenic molecular variants

**Figure 10:**
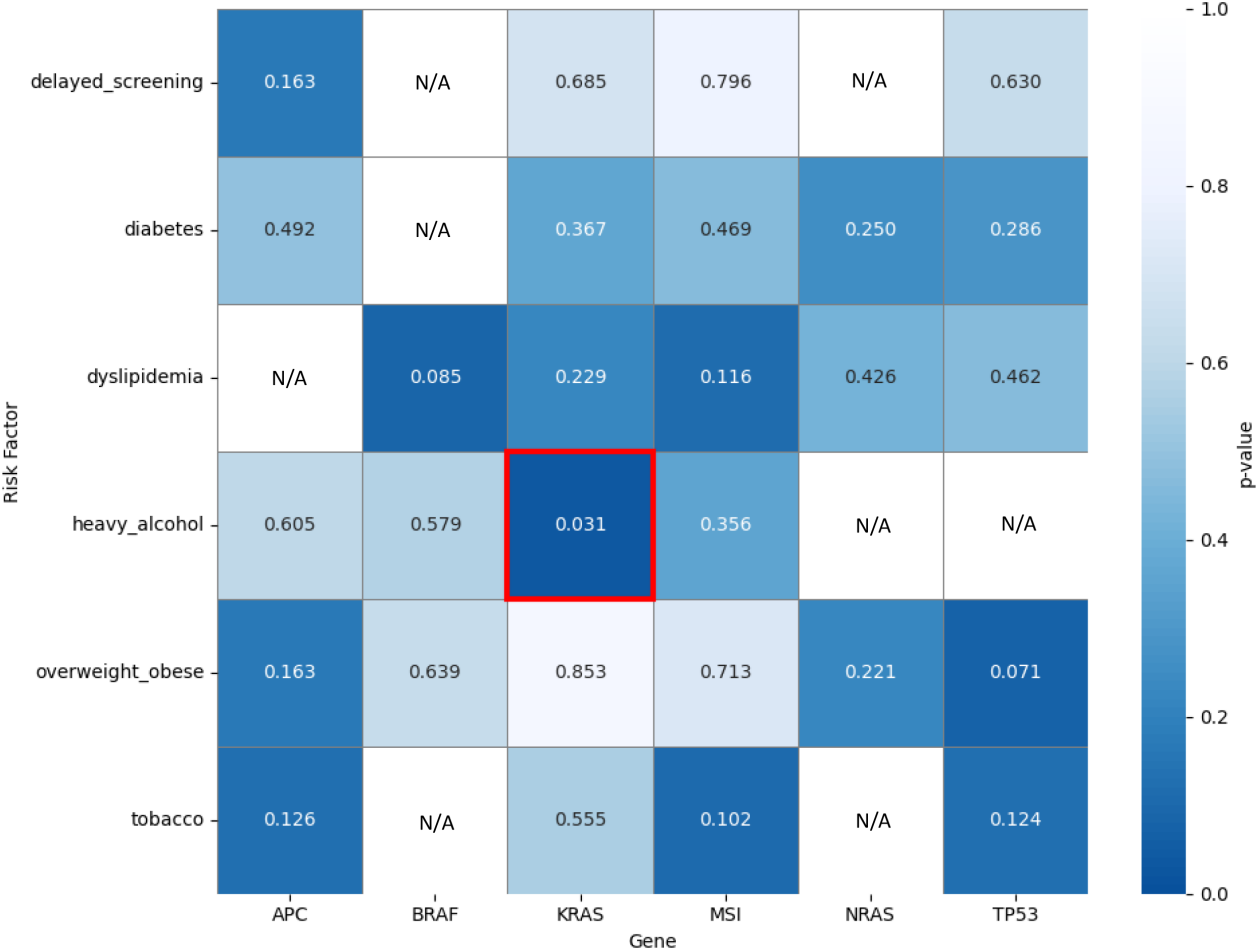
Comparing associations between tumor molecular alterations and colorectal cancer risk factors. The red box reflects a statistically significant finding prior to FDR correction.

Lastly, we compared the rate of microsatellite instability and tumor-specific KRAS, NRAS, BRAF, APC, and TP53 mutations among patients that had various CRC risk factors. Specifically, we compared the presence or absence of these molecular variants with the presence or absence of delayed screening, type 2 diabetes, dyslipidemia, heavy alcohol use, elevated BMI, and tobacco use. A Fisher’s Exact Test was used to determine if any one risk factor was significantly associated with the incidence of a particular mutation in our patients. Notably, across all age groups, it was shown that heavy alcohol use was associated with a higher prevalence of tumor-specific KRAS mutations (p = 0.031; OR 5.83; 95% CI 2.23-15.25) (**Figure 10**). Also noted in **Figure 10**, there appeared to be a non-significant association between tumor-specific TP53 mutations in patients who were overweight or obese, and tumor-specific BRAF mutations in patients with dyslipidemia (p = 0.071, and p = 0.085, respectively) (**Figure 10**). However, when using a Benjamini–Hochberg false discovery rate (FDR) correction on these data points, none of the three mutation-risk factor associations were found to be remotely close to statistical significance, with each having a corrected p-value of p = 0.504.

## Discussion

One of the primary objectives of this study was to attempt to identify differences between CRC patients 30–49 years of age and CRC patients ≥ 50 years of age. As the current guidelines recommending CRC screening at the age of 45 were updated in 2023, we wanted to ensure that the patients that we evaluated in this study were diagnosed prior to this revision. Despite not showing that younger patients were significantly more likely to present with late-stage disease compared to older patients, we believe that the association between younger patients and more advanced cancer in our data may support a clinically significant difference between these groups if examined further in a population-based study. However, this observation in our data should only be considered hypothesis-generating given the very small number of patients in our group aged 30–39 (n = 6). Such an observation is consistent with findings from other recent studies, which have demonstrated that younger patients < 50 years of age are significantly more likely to present with late-stage disease (19–22). Contributing factors to this trend appear to be multifactorial, with some even being due to underlying medical bias. Expectedly, younger CRC patients were significantly more likely to have a family history of CRC, known hereditary cancer syndrome, or suspected hereditary cancer syndrome (19).

While 27.6% of our patients aged 30–49 had a personal history of cancer, a family history of CRC, or otherwise strong family history of malignancy, this was not significantly different when compared to other age groups in this study. It has also been documented that younger patients are significantly more likely to present with distal colon and rectal malignancies compared to their older counterparts (19, 20, 23). Likewise, our patients aged 30–49 overwhelmingly presented with primary tumors from the sigmoid colon and rectum proportionally when compared to patients aged ≥ 50. Lower health literacy and socioeconomic status also appear to disproportionally affect younger patients, as they are more likely to ignore disease symptoms and have poor access to healthcare due to lack of insurance and the cost of healthcare (20, 21, 24). However, the most concerning contributing factor is physician oversight of red flag symptoms in favor of benign diagnoses due to the age of the patients (20, 21). This in combination with the aforementioned social factors contribute to late-stage disease via significant delays in screening and diagnosis—taking on average 1.4-times as long to reach diagnosis compared to older patients, which can translate to an overall delay of nearly 2 months, or up to 2 years in some patients (19–21, 25, 26).

Given the very small number of patients in our single-center retrospective study who were aged 30-49, repeat evaluation of CRC patients less than 50 years of age should be performed with better study design and ideally with population-level sampling. For a more in-depth look at this age group, it may be beneficial to separate this age group into those aged 30–44 and those aged 45-49, to examine if there is a difference in outcomes between these subgroups due to recommendations for routine screening to now begin at 45 years of age—with added consideration of patients who received clinically indicated screening at a younger age. Due to the concerning findings regarding CRC incidence among younger patients in recent literature, it would not be surprising to see recommendations for CRC screening in patients who are 40 years old within the next decade. In the meantime, promoting patient education on CRC risk factors and warning signs by clinicians in patients < 45 years of age will undoubtedly assist with improving outcomes in this group.

In our study, patients who identified as Black in our study had the lowest rate of screening delays, in addition to a comparable stage at diagnosis and outcome to patients who identified as Non-Hispanic White. While not significant, this is an interesting finding as these patients have repeatedly been shown to have significantly worse screening compliance, disease stage at the time of diagnosis, and poorer outcomes compared to Non-Hispanic White patients (27–29). Additionally, even with our modest sample size, we identified that our “Other” racial group was significantly more likely to present with more-advanced disease at the time of diagnosis compared to Non-Hispanic White patients and Black patients. While our data suggests that patients of all races received equitable healthcare at our single-center as shown by their overall similar outcomes based on disease stage, patients with more advanced-disease were found to have significantly worse outcomes as expected. Due to our study design, it is unclear if these findings would be consistent upon adding patients from additional facilities or on the regional population level. However, it should be considered that this correlation implies that patients in the “Other” group from our study may have disproportionately higher morbidity and mortality compared to Non-Hispanic White, and Black patients in our clinic.

Unfortunately, it is unclear if this trend with these patients is due to a lack of healthcare access, health education, or a lack of trust in the healthcare system. However, patients in this “Other” group were noted to have the highest rate of screening delays compared to Non-Hispanic White, and Black patients. Although, this group was not too dissimilar from the Non-Hispanic White group—both of which presented with a higher rate of screening delays compared to our Black patients. While this finding was not statistically significant, it is likely of clinical significance for this clinical environment, and a greater emphasis on the importance of routine screening in addition to more access to screening opportunities for these patients may improve disease burden over time. This is especially important for the historically under-screened groups which, beyond multiracial patients, likely comprise the “Other” racial group in this study—namely Hispanic, Asian, and American Indian patients. Hispanic patients in particular report an often significantly lower rate of CRC screening compared to other groups, with an overall reduction between 9.6% and 33%—and even lower rates observed in patients with limited English proficiency (30–33). Patients who identify as either Asian or American Indian have also been noted to have significantly lower CRC screening rates compared to other racial groups, often with screening rates comparably as low as Hispanic patients (32, 33). On a national level, it appears that patients across nearly all racial backgrounds have been noted to have a significant improvement in screening compliance from 2014-2020, with the exception of Asian patients (33).

Perhaps contrary to initial expectations, it was noted that our patients without metabolic risk factors for CRC—specifically dyslipidemia and diabetes—presented with more advanced disease than patients with either conditions alone, or with both comorbidities. While we strongly believe that this result does not imply that patients with these known risk factors are less likely to develop CRC than those without them both at the level of this clinic and beyond, it is important to try to identify why we observed such a result to such a significant degree. Aside from the fact that this comparison did not control for confounding lifestyle risk factors, unknown genetic risk factors, or other unmeasured environmental risk factors, another possibility is that this paradoxical result may be related to variances in healthcare visit frequency. This phenomenon, known as surveillance bias, relates to the idea that patients who have comorbid risk factors for malignancy are followed more closely by healthcare clinics and hence are more likely to be screened, and screened earlier than patients with fewer or no risk factors due to less-stringent follow-up (34–36). Another related explanation is that of access bias, which is a phenomenon in which certain patients have greater access to healthcare services than other patients, which may imply that patients either have limited access to healthcare professionals and may appear healthier due to lack of diagnostic testing, or that conversely unhealthier patients may have greater access to the healthcare system for any number of reasons. These are distinctly antithetical to the healthy patient bias, which proposes that patients who participate in cancer screening are more likely to have fewer chronic diseases or comorbidities due to a greater sense of health consciousness, and therefore identify disease sooner than less-healthy patients who may not follow-up with their healthcare providers as frequently. Regardless of the underlying reason for this artifact, this finding does not imply a protective effect of metabolic disorders, as the presence of one or more of these comorbidities are clearly correlated to an increased risk of developing CRC as demonstrated extensively throughout literature. Future evaluations of this patient population at either the clinic or regional level would likely benefit from analysis comparing proxies of patient healthcare utilization such as visit frequency, insurance type, and the presence or absence of a primary-care physician, in addition to proxies of patient socioeconomic status such as occupation, or insurance type as previously stated. Lastly, as the majority of our patients had at least one metabolic and one lifestyle risk factor, a comparison of disease stage for patients with only diabetes, dyslipidemia, elevated BMI, tobacco use history, or heavy alcohol use as singular risk factors would likely not have been feasible due to insufficient patient counts in each cohort, which may not have been an issue in a multi-center or population-based study. Thus, such an analysis was not included in this study. An argument could be made that such an issue can be seen in our comparative analysis of lifestyle risk factors with the group of patients who were both overweight/obese and reported heavy alcohol use. However, we felt that this was necessary to use this solitary, small group to comprehensively investigate the effect of multiple comorbidities on the presenting stage of disease.

While the importance of considering the role of patient demographics and modifiable risk factors in the development of CRC cannot be understated, we were particularly interested in evaluating tumor-specific molecular trends in this region and how they differ between patient age groups. In addition to examining tumor-specific mutations commonly associated with CRC, we also attempted to identify any reported variants of unknown significance appeared more frequently in CRC patients aged 30–49. Across all patients aged 30-49, only one gene was identified to appear with variants in tumor samples from more than one patient—SMAD4. Mutant variants of this gene appeared in tumors from three patients in this group, and in six patients from our ≥ 50 group. Unfortunately, when comparing the frequency of these variants using a chi-square analysis, we found that the rate of mutations in this gene in tumors from younger patients was not significantly different than tumors sampled in older patients. We identified that mutant variants of TP53 and APC found in tumor samples were significantly associated with late-stage disease compared to their wild-type variants. Such a result was not observed when comparing the mutational status of KRAS and NRAS in tumor samples from our patients. It has been well documented that APC mutations have a high prevalence in CRC—with either germline or somatic mutations present in as many as 80% of such tumors, it is regarded as a key component of CRC tumorigenesis (9, 37–39). Currently, data regarding the role of APC mutations in patient outcomes is mixed, with most studies identifying no clear relationship (37, 40).

However, it has been suggested that several subtypes of CRC with mutated APC have been associated with an intermediate prognosis (37). Furthermore, studies investigating the location of mutations within APC suggest that patients with metastatic CRC that have variable outcomes. One study noted that patients who had tumors with APC mutations that led to truncation of the protein ≤ 1256 amino acids saw a significantly worse overall survival; while another study suggested that patients who had tumors with mutations affecting C-terminal amino acids had a higher overall tumor mutational burden and worse overall survival (41, 42). Another recent study suggests that APC status in CRC patients has an effect on the efficacy of immunotherapy, as patients with tumors that have mutated APC appear to respond poorly to immunotherapy and have worse outcomes overall when compared to patients with tumors that have wild-type APC (38). Due to the importance of APC mutations in kickstarting CRC, this significant finding may be more related to overall mutational burden as every tumor sample identified to have an APC mutation in our study was also identified as having at least one, or multiple, KRAS, NRAS, BRAF, or TP53 mutations.

TP53 mutations are also commonly found in CRC, and have been shown to be significantly more common in left-sided tumors than right-sided tumors (43). While mutated TP53 is also not classically viewed as a prognostic factor for CRC, the presence of mutations to the L3 zinc-binding domain has been shown to correlate to reduced survival in patients with left-sided tumors, and gain of function mutations has been shown to significantly shorten overall survival in patients with highly-methylated late-stage disease (43–45). Interestingly, patients aged 30–49 in our study were also significantly more likely to present with tumors that had KRAS mutations and TP53 mutations than patients aged ≥ 50. Such trends have also been previously reported in literature; however tumor molecular factors that also appear to play a role in CRC incidence for younger patients include the presence of APC and BRAF variants (23). The presence of mutant TP53 in this age group is relevant, as these patients overwhelmingly presented with distal colon and rectal primary lesions, which is congruent with the aforementioned trends that have been routinely documented within literature surrounding CRC.

Specifically, within this group of patients, we believe the increased frequency of these tumor-specific mutations may be multifactorial as it relates to subsequent findings regarding the influence of modifiable patient risk factors on tumor molecular alterations. Firstly, prior to multiple testing correction, we identified that heavy alcohol use may be associated with a higher prevalence of tumor-specific KRAS mutations across all age groups. The correlation between chronic alcohol use and an increased prevalence of tumor-specific KRAS mutations in gastrointestinal cancers has been documented previously—specifically in patients with pancreatic cancer and CRC (46–48). While our patients aged 30–49 did not report excessive alcohol use during their visits to clinic, it is important to recognize that the accuracy of self-reported drinking behavior is likely negatively impacted, particularly in this group, due to the negative stigma associated with chronic alcohol use. This is supported by the fact that in our study, only 9.6% of patients reported having ≥ 3 standard drinks per day, whereas Lucas County has a self-reported excessive alcohol use rate (defined as ≥ 8 standard drinks per week for women and ≥ 15 standard drinks per week for men) of 20% in 2023 (49, 50). Notably, this volume of alcohol consumption is higher than the threshold used for our study, as we utilized classifications based on the shocking results from the 2020 study on the global burden of cancer attributable to alcohol use by Rumgay et al. which noted significantly higher rates of cancer among patients who reported heavy drinking (20–60 g per day; 2–6 standard drinks) and risky drinking (> 60 g per day; > 6 standard drinks) (51). Furthermore, in the context of the patient population that fits within this the 30–49 age group from our study, we were predominantly examining Millennials and the latter portion of Generation X. Millennials have been shown to consume alcohol at a much higher rate than previous generations, and are more likely to binge drink compared to members of Generation X or Baby Boomers (52). We believe that this potential correlation between the increasing incidence of CRC in patients aged 30–49 and excessive alcohol use as suspected by our findings relating heavy alcohol use to tumor-specific KRAS mutations is potentially novel and hypothesis-generating. For this reason, we believe it should be examined more closely using either a multi-center or population-based study design with an improved system for monitoring and reporting alcohol use.

Secondly, we also identified an association between elevated BMI and an increased prevalence of tumor-specific TP53 mutations. While this finding was not statistically significant, the presence of tumor-specific TP53 mutations in obese patients has been previously documented as a significant prognostic marker of poor outcomes due to its correlation with late-stage disease (53, 54). Interestingly, the presence of tumor-specific KRAS mutations in CRC patients was also found to be predictive for the presence of tumor-specific TP53 variants as well (54). A similar trend towards significance was observed in our data which related dyslipidemia in patients to tumor-specific BRAF mutations. When looking at our patients, those aged 30–39 and 40–49 had higher rates of elevated BMI compared to other age groups, with 83.3% and 82.6% being either overweight or obese, respectively. The next closest group pertaining to elevated BMI was our 70–79 age group, with 80.3% being overweight or obese. Notably, the 2022/2023 Lucas County Community Health Assessment identified that 32% of the population was overweight and 43% were obese, whereas the state of Ohio reported that 33% of the state population was overweight and 36.4% were obese (55, 56). These trends likely reflect inadequate physical activity and poor diet, which are characteristic of this part of the Midwest, as evidenced by its increased prevalence of obesity, metabolic syndromes, and metabolic syndrome severity (57, 58).

Despite our interesting findings, this study was not without its limitations. Firstly, we acknowledge that for a study initially looking to evaluate cancer patients on a regional level, the quantity of patients that were enrolled in this project (n=230) is insufficient to truly elucidate larger population trends by skewing statistical outcomes as either significant or non-significant when true underlying trends may be vastly different. For that reason, this study should be interpreted as a clinic-based study and our findings should be considered hypothesis-generating for larger, future studies rather than reflecting actual population-level trends. As an example, the Benjamini–Hochberg FDR analysis of the findings noted in **Figure 10** indicating that the statistically significant correlation between alcohol use and the presence of tumor-specific KRAS mutations in our dataset is likely not representative of a real trend if re-examined in a future population-based study. Additionally, at the studied clinic, tumor molecular testing was performed based on clinical indication. As only 63.0% of our patients had testing performed, our findings may be skewed to preferentially reflect these clinician-selected patients rather than providing a true representation of the molecular profile of all patients in this clinic. This also restricted key analyses for use in comparing CRC patients aged 30–49 to the remaining cohort, especially with respect to MSI/MMR status. Furthermore, our small sum of patients aged 30-49—with only 6 patients being in the 30–39 age group—likely had a negative impact on the quality of our statistical analyses that evaluated this group and compared it against our other age groups. Not to mention, no patient genetic testing was available within the EMR—only tumor-specific Tempus sequencing reports were available. Due to this limitation, no definitive comparison between patients in our study with somatic mutations and patients in our study with germline mutations could be made.

Furthermore, the demographic profile of our patients in this study was not entirely reflective of the population within the studied region. This is because the population of the greater Toledo area (including Toledo, Sylvania, Bowling Green, Maumee, Perrysburg, and Oregon) is 563,527 based on the 2020 census, and consists of 70.5% Non-Hispanic White, 15.7% Black, and 13.8% Other (including 0.3% American Indian and Alaska Native, 1.8% Asian, < 0.1% Native Hawaiian and Other Pacific Islander, 2.2% Other, and 7.2% Multiracial), in addition to the sex of the population being 48.6% male and 51.4% female (59, 60). Our analysis was further limited by the inability of the electronic medical record (EMR), OncoEMR, to display patient races beyond “Non-Hispanic White,” “Black,” “Hispanic,” and “Other,” which in combination with our pool of patients being over-represented by Non-Hispanic White patients and patients who identified as male, may have limited our analysis to identify the needs of marginalized groups. Future analysis would ideally utilize an EMR that has inclusion of more heterogeneous groups to reduce the risk of ecological fallacy, because the consequence of reducing these distinct groups into one conglomerate as above likely masked intragroup disparities that a dataset utilizing another EMR may have been able to identify. It is also unclear what role the COVID-19 pandemic played in reducing rates of CRC screening in our patient population, if any. However, it is notable that our patients ≥ 50 years of age had an overall up-to-date screening rate of 80.1%, which is the national goal for CRC screening, which is impressive when compared to the national up-to-date screening rate of 69.7% for patients aged 50–75 in 2020 (61).

This brings us to our next concern, the EMR used for data acquisition. As mentioned previously, OncoEMR limited our ability to further classify patients based on their race for an improved demographic analysis of our patients. Other concerns with this EMR include; inconsistencies in charting between patients, and the inability to review notes from providers outside of the Department of Hematology/Oncology at Toledo Clinic. Regarding chart inconsistencies, many of the integral documents that were used in this study, such as patient tumor sequencing reports from Tempus, were not digitally reported and were instead scanned into the chart. While the conversion of physical documents to a digital format is an appropriate method for updating a patient’s chart, it is possible that variances in where these documents were uploaded, delays in uploading such documents, or the absence of digital copies of these documents reduced the ability to include more data points in this study. For example, we were unable to compare tumor PD-L1 expression levels between our patients due to the testing either being unavailable from not being included in the scanned documents, or from not being performed. Additionally, there is a reasonable likelihood that patients who were believed to be lost to follow-up may have succumbed to their disease and became deceased instead—particularly in patients who were ≥ 80 years of age. However, if this information were not provided to the clinic or updated in the patient chart, it is not possible to determine patient status accurately. This oversight could have also potentially been minimized if provider notes and other updates from outside of the Department of Hematology/Oncology at Toledo Clinic could have been obtained and read. Additionally, while patient past medical history was available in this EMR, it does not appear to be carried over from outside EMR services, meaning that new diagnoses, or potentially predisposing conditions such as inflammatory bowel disease (IBD), Lynch Syndrome, or Familial Adenomatous Polyposis (FAP) may not have been reported accurately and may have ultimately limited our ability to evaluate these patients. Specifically, we were only able to identify three patients with IBD and three patients with confirmed Lynch Syndrome.

Future studies would likely be improved by broadening demographic data to better evaluate more ethnic backgrounds as mentioned previously, and to also increase the number of clinics or hospitals sampled from. While we believe that the total population of patients at Toledo Clinic provide reasonable coverage of patients from the Northwest Ohio region—with most patients being from Wood and Lucas counties, but also included several patients from Fulton, Henry, Ottawa, and Sandusky counties—our findings are again restricted to clinic-based associations that most likely do not represent true, actionable population-level trends of the Northwest Ohio region. This is ultimately due to the design of the study itself, as the retrospective single-center design significantly limits the generalizability of our findings as a consequence of the bias generated from the restricted pool of patients we examined. Furthermore, this study failed to consider the potential for the exclusion of patients who had surgically cured early-stage cases and were not referred to the outpatient oncology clinic. Additionally, since we only examined one clinic in our study, there is also a high likelihood of referral bias in which the specific factors that lead to a patient being referred to one clinic versus another or none at all—including socioeconomic factors, patient insurance, patient age, disease severity, referring provider bias, and geographic proximity to the clinic itself—also limit the generalizability of our findings to the greater area. This is especially relevant given the presence of both the Promedica and University of Toledo health networks locally, and the proximity to larger tertiary centers including The Ohio State University, the University of Michigan, the Cleveland Clinic, and the Karmanos Cancer Institute for particularly advanced or complicated cases. A number of the patients who ultimately transferred their care ended up selecting one of these aforementioned facilities to continue receiving evaluation and care, further affecting the ability to analyze outcomes among other variables. In the context of this study, referral bias also likens itself to survivorship bias when considering immediately life-threatening or otherwise complex cases, as it is highly probable that certain cases in the community affecting patients of any age ultimately pursue quality of life measures over cancer-directed therapy.

It is for these reasons that we believe that our findings should be utilized as a hypothesis-generating tool for future analyses looking to more-accurately represent the population of this region to elucidate actionable trends for public health initiatives. Such studies would ideally include a larger pool of patients who are ideally proportionally distributed across each of the counties in this region based on their population density. For instance, in this region, two other hospital networks exist for oncology patients—the Promedica and University of Toledo systems, which would have not only increased the quantity of patient data to assess, but also would have broadened the geographical scope of this study to include patients from Hancock, Putnam, Paulding, Defiance, Williams, and Seneca counties. While these institutions were also contacted during the initial planning and data acquisition stages of this study, access to the tumor library data of either facility was unfortunately not able to be obtained. Additionally, as this was a retrospective chart review, it was simply not possible to identify and compare specific environmental risk factors that our patients were exposed to, including specific dietary components of our patients and possible occupational exposures to hazardous substances. For example, we ultimately were unable to assess patients who were veterans as a demographic category for two reasons. Beyond the fact that we were only able to identify five such patients, it was also difficult to assess which conflict they served in or where they trained, and ultimately impossible to determine if they saw time in combat. It is possible that any number of these untraceable, or underreported, risk factors contributed to the development of CRC in our patients.

## Conclusion

In conclusion, we believe that with this study we have produced a set of hypothesis-generating data to be utilized as a foundation for future studies evaluating CRC patients in the Northwest Ohio region. Overall, we believe that our clinic-based associations will contribute to the discussion of CRC in patients aged 30–49, both on a local and regional level. Our hope is that our findings will inspire further investigation on a larger scale in order to direct public health initiatives with the ultimate goal of further limiting disease burden and improving outcomes in our patients.

## Data Availability

The original contributions presented in the study are included in the article/supplementary material. Further inquiries can be directed to the corresponding authors.
